# Comment on “Argon I Lines Produced in a Hollow Cathode Source, 332 nm to 5865 nm”

**DOI:** 10.6028/jres.112.023

**Published:** 2007-12-01

**Authors:** Craig J. Sansonetti

**Affiliations:** National Institute of Standards and Technology, Gaithersburg, MD 20899

**Keywords:** argon, atomic spectroscopy, Fourier transform spectroscopy, wavelengths

## Abstract

Recent observations at the National Institute of Standards and Technology indicate that the Ar I wavenumbers reported by Whaling et al. [J. Res. Natl. Inst. Stand. Technol. 107, 149 (2002)] are systematically too large. To investigate the source of this problem, selected lines of Ar I and Ar II were remeasured in the same spectra used by Whaling et al. The measurements show that the Ar I wavenumbers of Whaling et al. are systematically shifted with respect to the Ar II wavenumbers previously reported by Whaling et al. [J. Quant. Spectrosc. Radiat. Transfer **53**, 1 (1995)] based on the same spectra. The Ar I wavenumbers can be corrected by a constant multiplicative correction of 0.999 999 933.

## 1. Introduction

In Ref. [1], Whaling, Anderson, Carle, Brault, and Zarem presented a comprehensive list of Ar I lines in the region 332 nm to 5865 nm as emitted from a hollow-cathode discharge with Ar buffer gas. The measurements were based on spectra retrieved from the archives of the 1 m Fourier transform spectrometer (FTS) of the National Solar Observatory (Kitt Peak). These spectra had previously been used by the same authors to measure lines of Ar II in this spectral region [2].

In recent work at the National Institute of Standards and Technology (NIST), 17 lines of Ar I ranging from 22163 cm^−1^ to 25315 cm^−1^ were observed in a Hg/Ar electrodeless discharge lamp (edl) with an FTS optimized for the ultraviolet [3]. The spectra were calibrated using precisely known lines of ^198^Hg [4]. For all 17 lines the NIST wavenumbers were smaller than the wavenumbers of Whaling et al. [1]. Pressure shifts due to differences in the Ar carrier gas pressure were investigated as a possible cause of the discrepancy but were not able to explain it.

In a separate set of experiments, the infrared spectrum of a Th/Ar hollow cathode lamp was observed with the NIST 2 m Fourier transform spectrometer [5]. The spectra were calibrated by Th lines measured by laser optogalvanic spectroscopy [6]. Approximately 600 lines of Ar I were measured. When these results were compared to Ref. [1], it was apparent that they also were smaller on average than the wavenumbers of Whaling et al. [1]. For lines associated with energy levels of high angular momentum or high excitation, the deviations were large and scattered. For several hundred low excitation lines, however, the NIST observations were smaller by a consistent shift of approximately 1 part in 10^7^. Based on these two sets of measurements in widely separated spectral regions, it appeared possible that the results of [1] might be high due to a systematic calibration error.

We have used Ar II wavenumbers from Ref. [2] in several experiments and have found them to be consistent with other generally accepted wavelength standards. It seemed interesting, therefore, to try to determine whether the Ar I wavenumbers from Ref. [1] and Ar II wavenumbers from Ref. [2] were consistent with each other. To investigate this question we retrieved three of the spectra used for the Ar measurements — Ti #7 (8/15/92), Cu #2 (4/17/83), and Ti #2 (11/19/85) — from the Kitt Peak Archives [7]. The spectra were analyzed using the interactive program Xgremlin [8]. Each line of interest was fit with a Voigt profile convoluted with the FTS instrumental function to obtain the wavenumber.

Ti #7 (8/15/92) covers the region of the NIST Hg/Ar edl observations. In this spectrum we measured the 28 Ar II lines recommended by Learner and Thorne for use as calibration lines [9]. From these lines we calculated a correction factor for the spectrum with an uncertainty of 2 parts in 10^9^, as shown in Table 1. We then measured the 17 Ar I lines that appeared in the NIST Hg/Ar observations and corrected their wavenumbers using the factor from Table 1. The results are shown in Table 2, where they are compared with the values from Whaling et al. [1]. The values from [1] are larger on average by the multiplicative constant 1.000 000 065 7(24). The combined standard uncertainty is calculated as the sum in quadrature of the standard error of the mean, representing the random scatter of the data, and a systematic component that propagates directly from the standard error in the calibration factor.

Cu #2 (4/17/83) covers a portion of the infrared in which many Ar I lines appear in the NIST Th/Ar hollow-cathode observations. In this spectrum we chose 20 lines of Ar II with high signal-to-noise ratio and good line symmetry for use in determining the wavenumber correction factor. The results are shown in Table 3. The scatter is significantly larger than for the recommended lines used in the previous spectrum, and two of the lines produce correction factors that are clear outliers. We excluded these lines, shown in italics in Table 3, in calculating the average. We then measured 20 Ar I lines that had high signal-to-noise ratio, good symmetry, and were associated with states of low excitation and low angular momentum. The wavenumbers for these lines were corrected with the factor from Table 3. The results are shown in Table 4. Again the results of Whaling et al. [1] are systematically larger. Neither the correction factor nor the ratio in Table 4 is determined as precisely for this spectrum as for Ti #7 because the number of significant figures given in Ref. [2] becomes a limiting factor; however, to a good approximation the results of [1] are again larger by a constant factor.

Ti #2 (11/19/85) covers the near ultraviolet to near infrared region between the two sets of unpublished NIST observations. In this region 18 Ar II lines are recommended for use as wavelength standards [2]. One of these lines was too weak to measure, but the remaining 17 were measured and used with the published wavenumbers from [2] to determine values of the correction factor, as shown in Table 5. The two longest wavelength lines produced correction factors that are in poor agreement with the other lines, and the overall scatter of the data is larger than in the other spectra. Since some of the lines recommended as standards were weak, an additional 20 lines were selected and measured based solely on their high signal-to-noise ratio and good symmetry. These lines produced correction factors with about the same average but a standard deviation 1.6 times larger than the original set; consequently, the average correction factor was calculated from the recommended lines, omitting the two outliers from the average. We then measured 20 lines of Ar I that were selected using the same criteria as for Cu #2 (4/17/83). The wavenumbers were corrected using the correction factor from Table 5. The results are presented in Table 6. As in the other two spectra the results from [1] are systematically larger than the new measurements.

Based on this sample of lines from three different spectra, which cover a wide range of wavelengths from the ultraviolet to the infrared, it is clear that the Ar I wavenumber scale in Ref. [1] is not consistent with the Ar II wavenumber scale in Ref. [2], despite the fact that Ar I and Ar II were measured in the same spectra. For each of the three spectra the scales differ by a multiplicative constant. The constant is defined most precisely for the spectrum Ti #7 (8/15/92), but the values for all three spectra agree within their uncertainties.

We have confidence that the wavenumbers for Ar II [2] are reliable. They have been widely used as standards for calibration of FTS spectra and have been shown to be on a consistent scale with independently determined standard lines of ^198^Hg I at a level of better than 2 parts in 10^8^ [10]. Therefore, we conclude that there must be a systematic calibration error in the Ar I results of Ref. [1].

On an empirical basis the spectra we have investigated suggest that all of the Ar I data can be corrected by reducing the wavenumbers by a single multiplicative factor. This factor is well-determined from the results of Ti #7 (8/15/92) to be 0.999 999 934 3(24). The factors from Cu #2 (4/17/83) and Ti #2 (11/19/85), 0.999 999 945 5(121) and 0.999 999 925 9(213) respectively, have larger uncertainties but are fully consistent with this value.

Most of the spectra used for Refs. [1] and [2] were originally calibrated with respect to Ar II lines of Norlén [11] and later shifted to a revised wavenumber scale based on molecular lines of CO [12] as described in [2]. It is striking that the ratio determined in the fourth column of Table 2 is in almost perfect agreement with the ratio between the wavenumber scales of Whaling et al. [2] and Norlén [11], which was determined in [2] to be 1.000 000 067(8). All of the Ar I wavenumbers in [1] are larger by this factor than their values in an unpublished list that was made available to several laboratories in the late 1990s [13]. This leads us to speculate that the systematic error in the published wavenumbers for Ar I [1] resulted from an inadvertent double correction of the wavenumber scale. We have communicated these results to Whaling, but he is not able to verify that a double correction actually occurred [14].

Our speculation is supported by a comparison of the deviations in the third columns of Tables 2, 4, and 6 for each line with the error that would result had the scale correction been applied twice. The average difference of these values for all 57 lines measured is just −0.000 01 cm^−1^. For 26 of the lines the difference is less than 0.000 05 cm^−1^, and the largest individual deviations are +0.000 19 cm^−1^ and −0.000 28 cm^−1^. These deviations are less than the reported uncertainty for all of the lines; thus the data are entirely consistent with a double correction of the scale.

## 2. Conclusion

Based on all of the evidence, we propose that the results of [1] be corrected by multiplying all wavenumbers by the factor 0.999 999 933. This factor is the inverse of the Norlén-to-CO scale correction. It is consistent with the corrections determined empirically from the spectra Ti #7 (8/15/92), Cu #2 (4/17/83), and Ti #2 (11/19/85). By making this correction, the Ar I wavenumbers of Ref. [1] will be put on the same scale as the Ar II wavenumbers of Ref. [2], which was derived from CO.

## Figures and Tables

**Table 1:** Determination of correction factor for the spectrum Ti #7 (8/15/92) from 28 Ar II lines.

Ar II Uncorrected (cm^−1^)	Whaling Ar II [2] (cm^−1^)	Correction Factor
19429.76452	19429.7694	1.000 000 251
19749.38149	19749.3873	1.000 000 294
19957.16070	19957.1662	1.000 000 275
20106.36850	20106.3743	1.000 000 288
20135.04095	20135.0466	1.000 000 281
20265.11937	20265.1249	1.000 000 273
20448.18949	20448.1949	1.000 000 264
20486.65005	20486.6556	1.000 000 271
20622.10647	20622.1121	1.000 000 273
20801.41733	20801.4230	1.000 000 273
20981.08382	20981.0895	1.000 000 271
21109.37593	21109.3817	1.000 000 273
21149.73502	21149.7409	1.000 000 278
21462.88349	21462.8892	1.000 000 266
21831.04007	21831.0458	1.000 000 262
21995.77800	21995.7838	1.000 000 264
22561.94887	22561.9551	1.000 000 276
22566.06057	22566.0667	1.000 000 272
22587.41164	22587.4176	1.000 000 264
22715.79393	22715.8001	1.000 000 272
22720.38659	22720.3928	1.000 000 273
22826.36872	22826.3751	1.000 000 279
22992.27461	22992.2808	1.000 000 269
23081.79760	23081.8043	1.000 000 290
23431.66861	23431.6744	1.000 000 247
23644.29776	23644.3037	1.000 000 251
25447.00142	25447.0076	1.000 000 243
26806.99465	26807.0020	1.000 000 274
	Average	1.000 000 270
	Standard Error	0.000 000 002

**Table 2:** Calibrated wavenumbers for selected lines of Ar I from the spectrum Ti #7 (8/15/92).

Ar I Corrected Ti #7 (8/15/92) (cm^−1^)	Whaling Ar I [1] (cm^−1^)	Deviation (cm^−1^)	Ratio Whaling/Ti #7
22163.1288	22163.1303	0.0015	1.000 000 0689
23007.6021	23007.6035	0.0015	1.000 000 0630
23059.7703	23059.7718	0.0015	1.000 000 0637
23069.2254	23069.2269	0.0015	1.000 000 0650
23248.7295	23248.7308	0.0013	1.000 000 0559
23400.7302	23400.7318	0.0016	1.000 000 0667
23432.9952	23432.9967	0.0015	1.000 000 0657
23471.0895	23471.0911	0.0016	1.000 000 0675
23798.9963	23798.9979	0.0016	1.000 000 0661
23812.3597	23812.3612	0.0015	1.000 000 0647
23853.7662	23853.7677	0.0015	1.000 000 0650
23855.5661	23855.5677	0.0016	1.000 000 0667
23905.9332	23905.9348	0.0016	1.000 000 0655
24007.5674	24007.5691	0.0017	1.000 000 0693
24039.8321	24039.8337	0.0016	1.000 000 0672
24718.4549	24718.4566	0.0017	1.000 000 0695
25315.8386	25315.8403	0.0017	1.000 000 0673
		Average	1.000 000 0657
		Standard Error	0.000 000 0008
		Uncertainty	0.000 000 0024

**Table 3:** Determination of correction factor for the spectrum Cu #2 (4/17/83) from 20 Ar II lines. The two lines shown in italics were excluded from the average.

Ar II Uncorrected (cm^−1^)	Whaling Ar II (cm^−1^)	Correction Factor
3312.75206	3312.7464	0.999 998 293
3336.55291	3336.5474	0.999 998 347
3405.89968	3405.8945	0.999 998 478
3423.09192	3423.0862	0.999 998 330
3441.05069	3441.0452	0.999 998 404
3469.59959	3469.5940	0.999 998 389
3495.53382	3495.5280	0.999 998 336
3519.86194	3519.8561	0.999 998 340
3562.07384	3562.0678	0.999 998 304
3580.41755	3580.4113	0.999 998 256
3612.33588	3612.3300	0.999 998 373
3617.29540	3617.2893	0.999 998 314
3620.50053	3620.4946	0.999 998 362
3695.22406	3695.2181	0.999 998 386
*3765.74583*	*3765.7387*	*0.999 998 107*
3807.75799	3807.7517	0.999 998 348
3878.80819	3878.8018	0.999 998 354
3906.18281	3906.1761	0.999 998 282
*4030.02928*	*4030.0201*	*0.999 997 723*
4031.86939	4031.8627	0.999 998 340
	Average	0.999 998 346
	Standard Error	0.000 000 012

**Table 4:** Calibrated wavenumbers for selected lines of Ar I from the spectrum Cu #2 (4/17/83).

Ar I Corrected Cu #2 (4/17/83) (cm^−1^)	Whaling Ar I [1] (cm^−1^)	Deviation (cm^−1^)	Ratio Whaling/Cu #2
3334.4969	3334.4971	0.0002	1.000 000 060
3415.2253	3415.2255	0.0002	1.000 000 059
3417.2965	3417.2967	0.0002	1.000 000 059
3435.4220	3435.4222	0.0002	1.000 000 058
3467.0372	3467.0373	0.0001	1.000 000 029
3504.0523	3504.0525	0.0002	1.000 000 057
3525.4135	3525.4137	0.0002	1.000 000 057
3573.3634	3573.3636	0.0002	1.000 000 056
3602.2958	3602.2960	0.0002	1.000 000 056
3619.6873	3619.6875	0.0002	1.000 000 055
3647.1137	3647.1139	0.0002	1.000 000 055
3663.9178	3663.9180	0.0002	1.000 000 055
3725.3641	3725.3642	0.0001	1.000 000 027
3757.6287	3757.6289	0.0002	1.000 000 053
3766.4387	3766.4389	0.0002	1.000 000 053
3828.0607	3828.0610	0.0003	1.000 000 078
3922.4007	3922.4009	0.0002	1.000 000 051
3978.9713	3978.9716	0.0003	1.000 000 075
4012.5895	4012.5897	0.0002	1.000 000 050
4171.3497	4171.3499	0.0002	1.000 000 048
		Average	1.000 000 0054
		Standard Error	0.000 000 0003
		Uncertainty	0.000 000 0012

**Table 5:** Determination of correction factor for the spectrum Ti #2 (11/19/85) from 20 Ar II lines. The two lines shown in italics were excluded from the average.

Ar II Uncorrected (cm^−1^)	Whaling Ar II (cm^−1^)	Correction Factor
*4776.24004*	*4776.2372*	*0.999 999 406*
*5833.12595*	*5833.1234*	*0.999 999 562*
7167.21529	7167.2141	0.999 999 834
7540.04974	7540.0487	0.999 999 862
8123.06894	8123.0686	0.999 999 959
8204.99534	8204.9945	0.999 999 898
8494.17032	8494.1690	0.999 999 844
8918.39741	8918.3950	0.999 999 730
9152.11655	9152.1152	0.999 999 852
9231.54287	9231.5434	1.000 000 058
9245.68391	9245.6829	0.999 999 890
9551.05683	9551.0551	0.999 999 819
9887.83232	9887.8323	0.999 999 998
10091.72748	10091.7258	0.999 999 834
10550.91915	10550.9176	0.999 999 853
10773.24592	10773.2439	0.999 999 812
11086.39442	11086.3923	0.999 999 808
	Average	0.999 999 870
	Standard Error	0.000 000 021

**Table 6:** Calibrated wavenumbers for selected lines of Ar I from the spectrum Ti #2 (11/19/85).

Ar I Corrected Ti #2 (11/19/85) (cm^−1^)	Whaling Ar I [1] (cm^−1^)	Deviation (cm^−1^)	Ratio Whaling/Cu #2
5929.5463	5929.5467	0.0004	1.000 000 065
6294.2542	6294.2547	0.0005	1.000 000 075
6521.6533	6521.6538	0.0005	1.000 000 073
6651.3143	6651.3147	0.0004	1.000 000 064
6858.0308	6858.0314	0.0006	1.000 000 081
7188.4110	7188.4115	0.0005	1.000 000 075
7338.7039	7338.7045	0.0006	1.000 000 084
7554.0590	7554.0597	0.0007	1.000 000 087
7910.1789	7910.1795	0.0006	1.000 000 076
8099.2837	8099.2843	0.0006	1.000 000 070
8403.4398	8403.4405	0.0007	1.000 000 089
8553.7326	8553.7333	0.0007	1.000 000 079
8717.8755	8717.8762	0.0007	1.000 000 084
8887.7592	8887.7599	0.0007	1.000 000 083
9178.2310	9178.2318	0.0008	1.000 000 084
9342.3739	9342.3745	0.0006	1.000 000 065
9541.1601	9541.1608	0.0007	1.000 000 068
9749.5939	9749.5947	0.0008	1.000 000 082
10217.4419	10217.4425	0.0006	1.000 000 057
10687.4305	10687.4309	0.0004	1.000 000 041
		Average	1.000 000 0074
		Standard Error	0.000 000 0003
		Uncertainty	0.000 000 0021
